# How soil type (gypsum or limestone) influences the properties and composition of thyme honey

**DOI:** 10.1186/s40064-016-3243-9

**Published:** 2016-09-27

**Authors:** Amelia Virginia González-Porto, Tomás Martín Arroyo, Carmen Bartolomé Esteban

**Affiliations:** 1 Laboratorio de Miel y otros Productos de la colmena, Centro Agrario de Marchamalo (CAR)-IRIAF, Junta de Comunidades de Castilla La Mancha, Camino de San Martín s/n, 19180 Guadalajara, Marchamalo Spain; 2 Departamento de Ciencias de la Vida (U.D. Botánica), Facultad de Biología, C. Ambientales y Químicas, Universidad de Alcalá, Madrid, Spain

**Keywords:** Pollen, Monofloral thyme honey, Soil type, Limestone, Gypsum, Honey properties

## Abstract

**Background and aims:**

The objective of this work was to determine the influence of the soil substrate on the characteristics and properties of a specific type of honey. As such, we analysed the features of a typical single-flower honey, thyme honey, produced in a specific Mediterranean region. *Thymus* is a genus of aromatic perennial plants that are native to Europe, North Africa and Asia.

**Methods:**

A total of 70 honey samples from hives situated on limestone (38 samples) or gypsum soils (32 samples) were studied. The physical and chemical properties of each samples were analyzed using standard assays.

**Results:**

Within the same geographical area and despite a similar thyme pollen content, we observed variation in the physicochemical, antioxidant and sensorial characteristics of monofloral honeys. The quantification of certain physicochemical parameters of the honey indicated these features were influenced by the soil type. Indeed, the soil type of the hives’ settlement area, limestone or gypsum, influences the conductivity, antioxidant capacity, colour and floristic composition.

**Conclusions:**

The present work demonstrates that soil type (gypsum or limestone) influences the characteristics of honey, potentially providing added market value to these products.

**Electronic supplementary material:**

The online version of this article (doi:10.1186/s40064-016-3243-9) contains supplementary material, which is available to authorized users.

## Background

Honey is a product derived from the nectar and sugar exudate of plants, material gathered and modified by honeybees, and stored in honeycombs. Floral nectar is a nutrient-rich solution offered by plants to their insect pollinators (Simpson and Neff 1983), and it is generally accepted that there is a co-evolutionary relationship between the sugar content of the nectar and the preference of some pollinators for certain sugars in their diet. The chemical content of the nectar is generally constant within a species (Nicolson 2007; Nicolson and Thornburg 2007) and flowers pollinated by long-tongued bees or butterflies tend to produce sucrose-rich nectar (Baker and Baker 1983; Nepi et al. 2010).

It is well known that species of the Lamiaceae (Labiatae) family are mainly pollinated by bees, even though they are visited by a relatively wide spectrum of insects (Dommée et al. 1978; Brabant et al. 1980; Morales 1986; Rolland 1999; Arroyo and Andres 2002). This family in general, and the genre *Thymus* L. in particular (*Thymus loscosii* Willk, *Thymus vulgaris* L., *Thymus granatensis* Boiss, *Thymus arundanus* Willk), produce a nectar rich in phenylalanine and sucrose. The ratio between these two compounds makes this nectar more attractive to bees, thought to reflect the co-evolution between these plants and their main pollinators (Baker and Baker 1983; Petanidou et al. 2000, 2006; Nepi et al. 2010).

Nectar production and its characteristics may fluctuate considerably in response to sometimes subtle changes in the environment, such as wind, temperature, soil moisture, or even the position of the flower on the plant and pollinator activity (Bertsch 1983; Hiebert and Caldera 1983; Pleasants 1983; Devlin and Stephensen 1985; Wilsen and Agren 1989; Belmonte et al. 1994; Gillespie and Henwood 1994; Torres and Galetto 1998). Floristic composition is a determinant factor in a honeys chemical content (Aazza et al. 2014; Karabagias et al. 2014a, b; Yang et al. 2014), clearly influencing its sensory characteristics. Indeed, the composition and properties of honey vary mainly in function of the floral sources utilized by bees (Moar 1985; Terrab et al. 2004; Dong et al. 2013; Lazarevic et al. 2013; León-Ruíz et al. 2013; Panseri et al. 2013; Rios et al. 2014). The pollen content in honey is thought to be particularly effective in defining the spatial distribution of the plant species in the region of hives, and the pollen present in honey may predict well the vegetation in a region (González-Porto et al. 2013). The pollen and physicochemical properties of monofloral honeys have been the subject of numerous studies (Persano Oddo and Piro 2004; Piazza and Persano Oddo 2004; Naab et al. 2008; Sabo et al. 2008; Makhloufi et al. 2010; Escuredo et al. 2011; Aloisi et al. 2013; Alves et al. 2013). In some studies, the physicochemical values or the pollen spectra obtained from thyme honeys has been correlated with the geographical origin of the honey (Alissandrakis et al. 2007; Karabournioti et al. 2009; Karabagias et al. 2014c). Thus, it is crucial for local beekeepers and their associations to produce honey with a geographically defined label of origin.

In the present study we have analysed samples of monofloral thyme honeys from the same region and the same harvest period (Alcarria region, Spain). In the territory where the beehives are located, there is little variability in altitude and climate. Thus, the physicochemical differences observed in these honeys should be due to the diversity of the flora (Anklam 1998; Terrab et al. 2002a, b; Acquarone et al. 2007; Alvarez-Suarez et al. 2010) and the local soil diversity. Floristic diversity depends on the soil and climatic conditions, and similarly, the sensorial characteristics and acidity of the honey are affected by the mineral salt content, a feature that also defines the honey’s conductivity. However, the properties of honey not only depend on the environmental conditions but also, on the extraction techniques (Feller-Demalsy et al. 1989; Bogdanov et al. 2008).

The objective of this work was to analyze thyme honey from the Alcarria region, separating these honeys into two groups related to the type of soil substrate, limestone or gypsum, based on a previous analysis. Based on the sensorial differences and differences in the pollen spectrum observed, we set out to determine the influence of the type of substrate on the antioxidant, physicochemical and organoleptic properties of the honey. We consider that it is of great importance to the beekeeping industry, food market and human health to be aware of the differences in the composition and properties of honey from distinct origins, not least for the possible therapeutic effects or pharmacoactive properties of the thyme honey produced.

## Methods

### Experimental design

#### Study area

The Alcarria region is a kind of plateau brought about by the rising of the Sistema Ibérico. Limestone and gypsum soils are common due to their origin below sea level in the Mesozoic Era. The Alcarria region covers about 2500 km^2^, of which 1473 km corresponds to Guadalajara, 650 km to Cuenca and 377 km to Madrid.

**Fig. 1:**
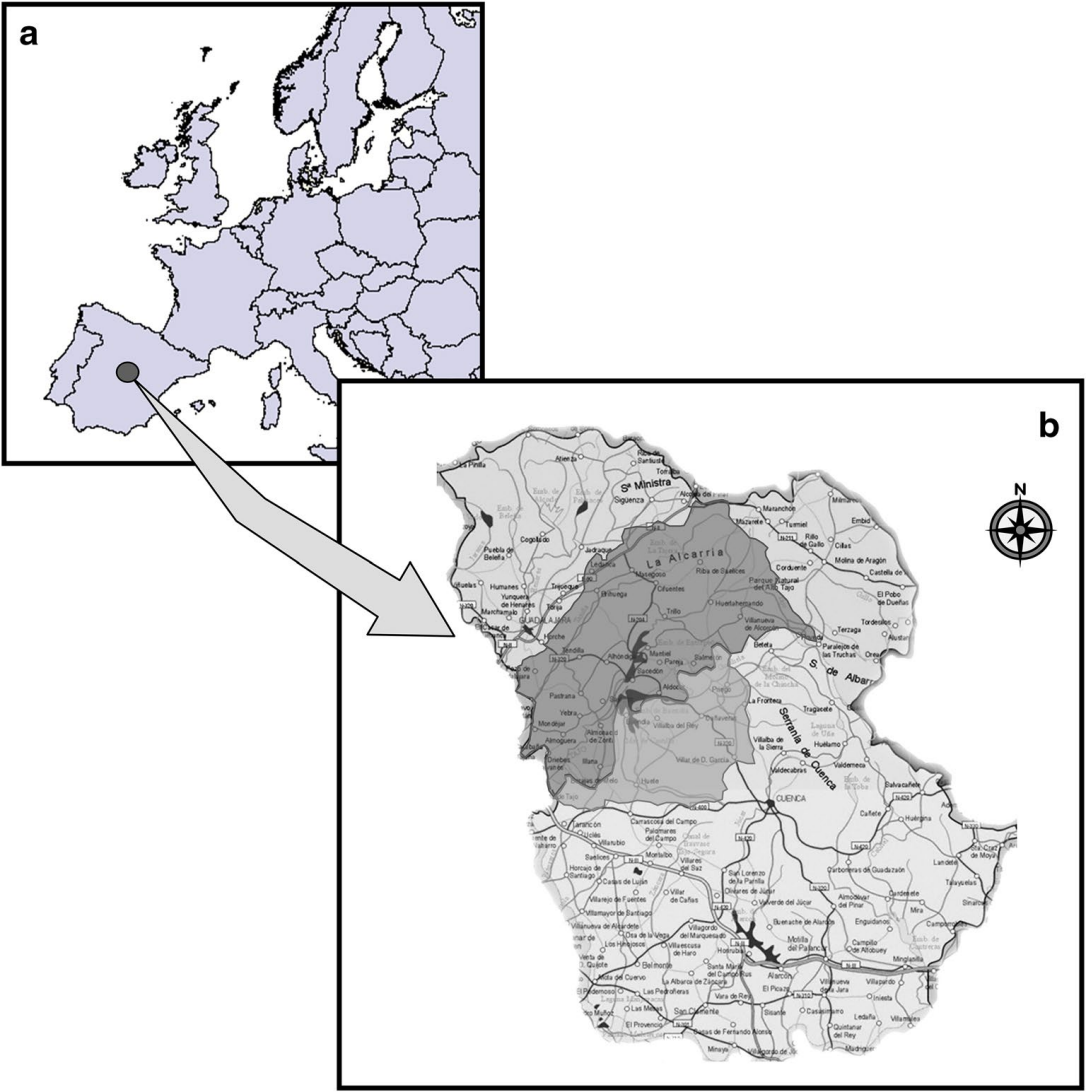
Map of the “Baja Alcarria” Region (Cuenca and Guadalajara provinces). **a** Location of Alcarria in Spain and Europe. **b** Location of the Alcarria area in the Cuenca and Guadalajara provinces. Image modified from the Topographic Map Spain. 1:25.000. Sheet 743, 2005. National Geographic Institute

The beehives studied here were located in a sub-region of the Alcarria region (Baja Alcarria) that includes the south of the province of Guadalajara, the Tajo River basin and the north of the province of Cuenca, between the Altomira formation and the Guadiana River basin (Fig. 1). The average altitude of the sites of the beehives is around 800 m, ranging between 600 m (Almoguera, Guadalajara) and 1075 m (Abia of Obispalia, Cuenca). Biogeograpically, the territory belongs to the Mediterranean Region, Mediterranean-Iberica-Central province, Castellana sub-province (Rivas Martínez et al. 1987; Rivas-Martínez 2004, 2007, 2008).

**Fig. 2:**
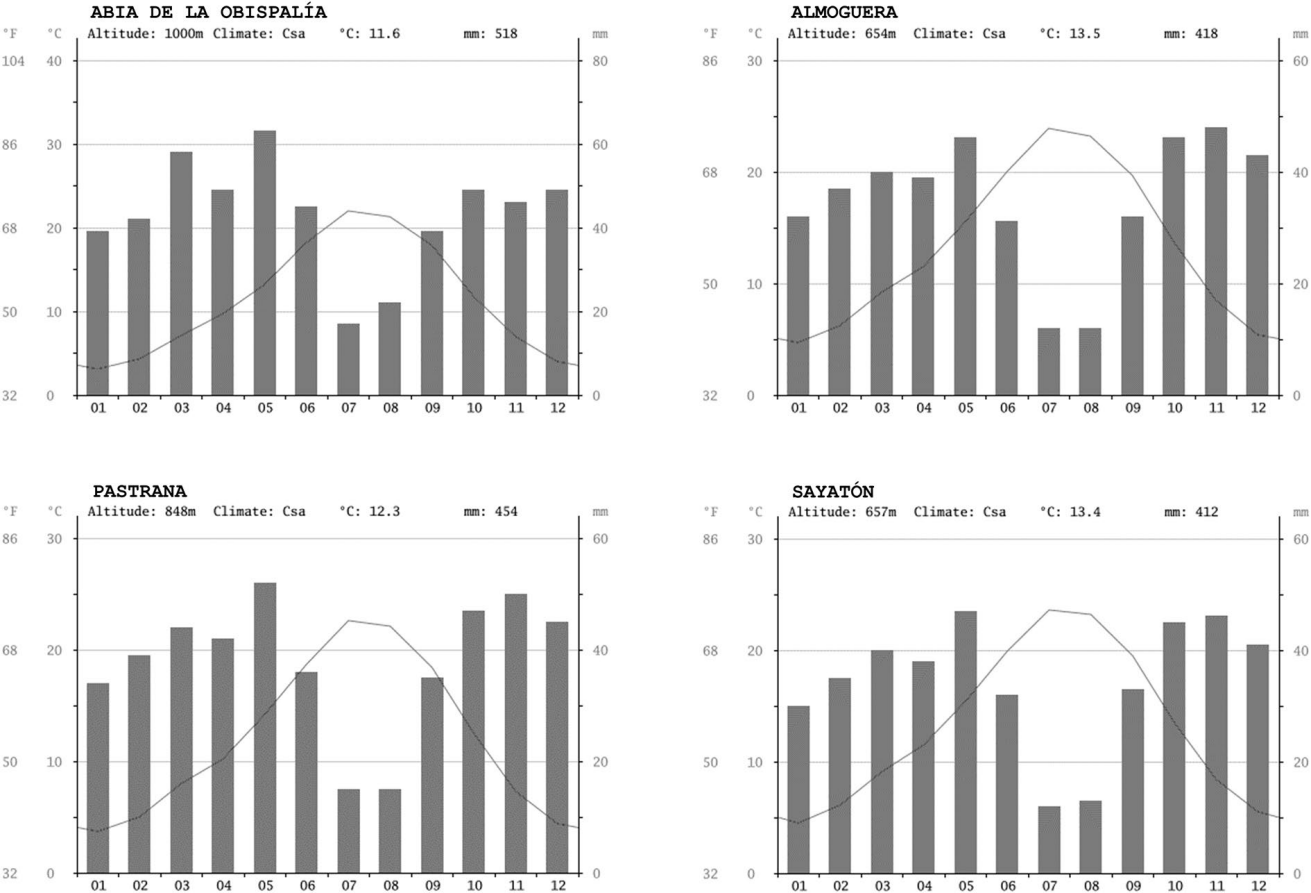
Climate mesomediterranean termothype, Mediterranean Region. Diagrams Bioclimatic models. Taken from Climate-Data.org. Information collected between 1982 and 2012

The dominant climate is identified as Meso-Mediterranean thermotype with a dry ombrotype (Aldeanueva et al. 1989; Papadakis 1966; Rivas-Martínez 2008). The variation in the annual average temperature between the areas of highest and lowest altitude is 1 °C and the difference in the annual precipitation is 100 mm. The territory suffers summer drought (Fig. 2).

**Fig. 3:**
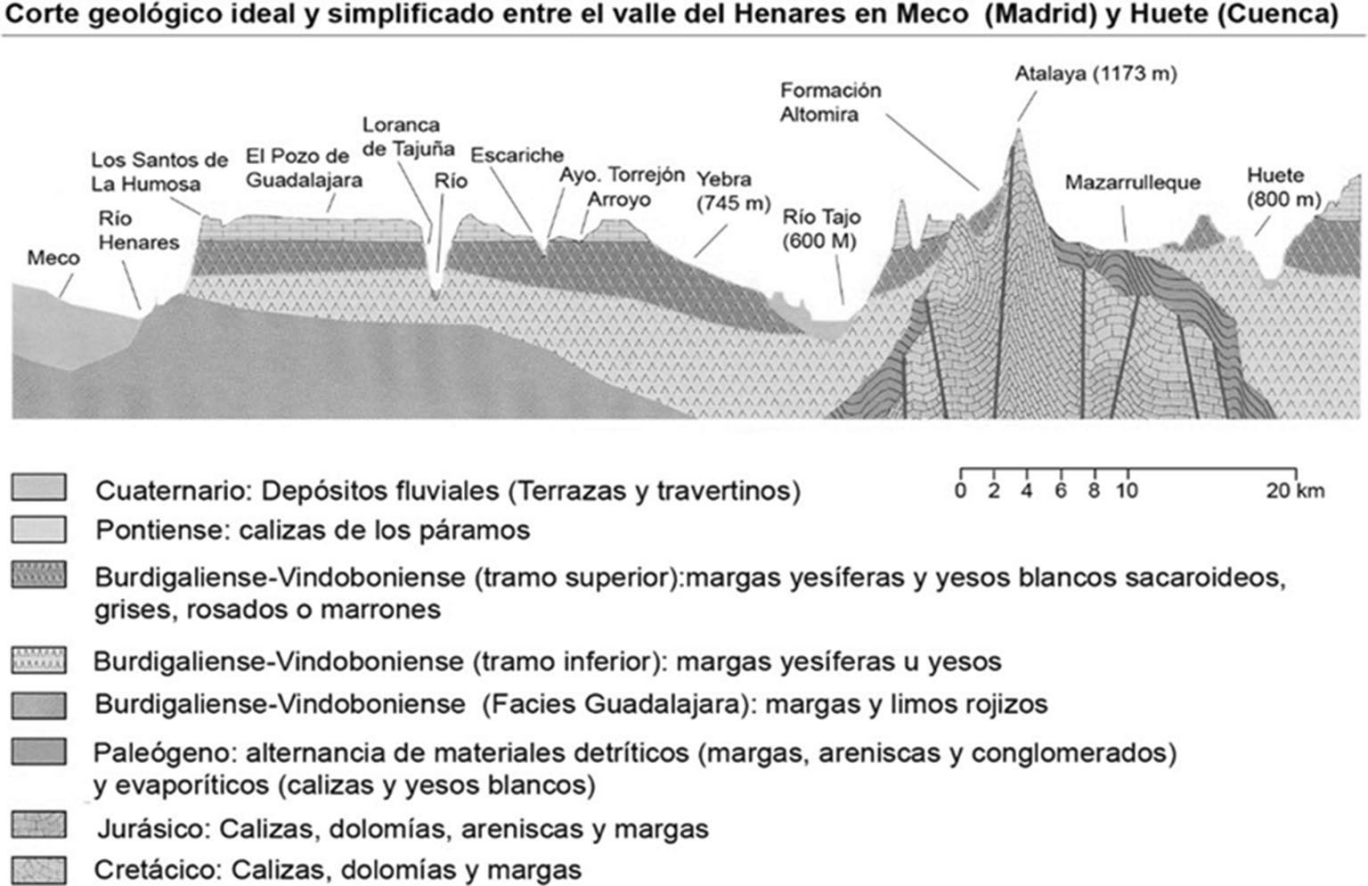
Cross-Section Geology between Santos de la Humosa (Madrid) and Huete (Cuenca). (Bartolomé et al. 2002)

With respect to the substrate, there is a succession of different types of gypsum in the Tajo and Major River basin that alternate with sandstones, marls and slimes. Generally, gypsum soils are located in the valley bottom whereas Jurassic and Cretaceous limestones appear at the top (Bartolomé et al. 2002; Rejos et al. 2011). This explains the soil variation in the territory (Fig. 3).

**Fig. 4:**
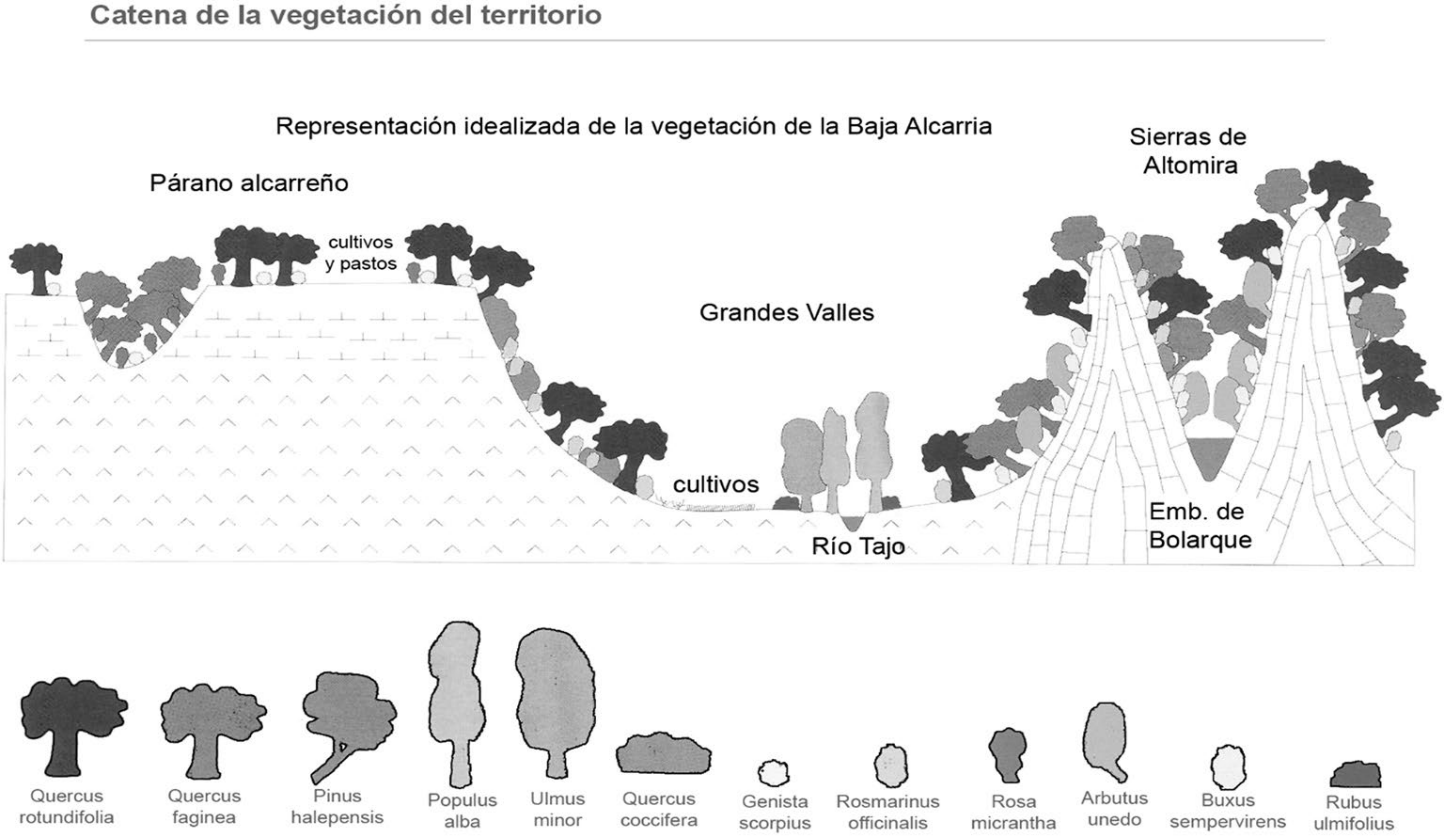
Catena idealized vegetation in “Baja Alcarria”. (Bartolomé et al. 2002)

As temperature and precipitation are similar throughout the study area, we deduce that the floral diversity is due to the edaphic heterogeneity. We studied the vegetation and flora on both types of substrate. The climax vegetation of the territory is the holm oak community (*Asparago acutifolii*-*Quercetum rotundifoliae*) and occasionally, in more humid areas and on the northern slopes of the valleys where there is thermal inversion, gall-oaks (*Cephalanthero longifoliae*-*Quercetum fagineae*). These gall-oaks predominate in the Celtibérico-Alcarreño sector of the Castellan sub-province. At present, due to the use of the territory, the most widespread vegetation is scrub, with different floristic compositions depending on the type of substrate: gypsum or limestone scrub (Fig. 4).

In terms of the flora, unique and endemic species appear on gypsum soils that are absent on the limestone soils: *Ononis tridentate* L (Fabaceae); *Cistus clusii* subsp *clusii* Dunal *Helianthemun squamatum* (L.) Dum. Cours., *Helianthemun syriacum* (Jacq.) Dum.Cours, *H. marifolium* subsp *conquense* Borja and Rivas Goday ex G. López (Cistaceae); *Thymus lacaite* Pau and *Teucrium pumilum* Loefl. ex L. (Lamiaceae = Labiatae); *Gobularia alypum* L. (Globulariaceae); *Artemisia herba*-*alba* Asso., *Centaurea hyssopifolia* Vahl, *Senecio auricula* Coss, *Launaea fragilis* (Asso) Pau and *Launaea pumila* Cav*.)* Kuntze (Compositae = -Asteraceae); *Brassica repanda* subsp. *gypsicola* Gómez Campo, *Isatis tinctoria* L, *Iberis amara L. Iberis saxatilis* subsp. *cinerea* (Poir.) Font Quer, *Moricandia moricandiodes* subsp *moricandioides* (Boiss) Heywood, *Lepidium cardamines* L and *Lepidum subulatum* L, *Eruca vesicaria* (L.) Cav. (Cruciferae = Brassicaceae), *Gypsophila bermejoi* G. López, *Gypsophila pilosa* Hudson, *Gypsophila struthium* subsp. *struthium* L. (family Caryophillaceae), *Herniaria fruticosa* L. and *Arenaria cavanillesiana* (Font Quer & Rivas Goday) Nieto Fel. (Caryophyllaceae).

The plant biodiversity and the floristic richness of this territory, situated in the centre of the Iberian Peninsula, makes it unique. The area has a number of endemic species at the national, regional and provincial level. Approximately 20 species in the area that grow on gypsum soils are included in various national and regional catalogues of endangered species, reflecting the region’s importance for biodiversity conservation (Mota Poveda et al. 2011).

### Location of the hives

In 2009, the hives on gypsum and limestone substrates within the area described were selected. Samples were collected in 2010, only monofloral thyme honey, and they were stored at −20 °C until analysis. All the honey samples were provided directly by the beekeepers and they have not been processed industrially.

Of the 82 samples initially analysed and in order to not distort the interpretation of the results, we selected those for which we could obtain information regarding all the parameters and those that represented good quality honeys. The tests performed were carried out in duplicate on all the samples in order to check their reproducibility. Accordingly, a total of 70 honey samples were studied: 38 from apiaries located at sites with limestone soil, 32 located on gypsum soils.

### Melissopalynological analysis

The honey samples were treated chemically with acidified water (10 % sulphuric acid) according to the harmonised method of Von der Ohe et al. (2004). A qualitative and quantitative count of the sediment recovered from 10 g samples revealed at least 300 pollen grains in each sample. The composition of the honey sediment was analysed under the microscope, and the pollen grains from each sample were identified and classified on the basis of the identification keys available at the C.A.R. honey laboratory (Valdés et al. 1987; Carretero 1989; Moore et al. 1991; Saa Otero et al. 1996), and the manual and digital pollen collections already available in the laboratory. The International Commission for Bee Botany (ICBB) recommendations were followed to classify the honey according to its floral origin (Louveaux et al. 1978), bearing in mind the minimum percentages of nectariferous pollen for monofloral honeys.

### Sensory analysis

Panel lists for the sensory descriptive analysis were selected from the external sensory panel of the Honey Laboratory of Centro Agrario de Marchamalo (Guadalajara, Spain). The 70 samples were tasted by a panel of 7 experts (20–60 years old) and the honeys were described according to defined sensory descriptors (Persano Oddo and Piro 2004) for European monofloral honeys. The definition of the main sensory analysis terms used can be found in ISO 5492 (1992). The parameters selected to describe European unifloral honey are visual, olfactory and taste. Tasting was carried out following the phases and methodology described by Piana et al. (2004), from whose data the corresponding fact sheets were developed. It was relevant to define which pollen grains contributed to the sensory variation among the honeys. Thus, a Partial Least Squares Regression (PLSR) model was established to elucidate possible relationships between the pollen grains and the sensory descriptors.

### Colour determination

The measurement of colour intensity was based on optical comparison using simple colour grading as defined by Pfund (Fell 1978) or Lovibond (Aubert and Gonnet 1983). Honey is generally marketed according to the Pfund colour scale, which is why Lovibond graders on a Pfund scale are currently used. Other more objective methods have also been used, such as the determination of all colour parameters through the CIELAB L*a*b* three-dimensional method (Aubert and Gonnet 1983; Ortiz Valbuena and Silva Losada 1990; Persano Oddo et al. 1995a). The CIELAB system is a reflection method (measuring geometry d80, illuminant D65, range 400–700 nm, observer 10^o^) carried out on a Hitachi model U-1100 spectrophotometer (L* lightness, a* chromaticity +red/-green, b* chromaticity +yellow/-blue, C*ab chroma, hab. tone).

### Determination of physicochemical parameters

Some physicochemical parameters were analysed using the Harmonised Methods of the International. Honey Commission (Bogdanov et al. 2004). Moisture level was determined by refractometry on an Abbé analogue refractometer, at 20 °C (Bogdanov et al. 1997). Electrical conductivity was measured at 20 °C in a 20 % (w/v) solution of honey (dry matter basis) in deionized water using a Radiometer CDM-83 conductimeter. The pH was measured potentiometrically at 20 °C in a 10 % (w/v) solution of honey in freshly boiled distilled water using an Eutech System pH meter (model XS PC510). The free acidity was obtained by plotting the neutralization curve titrated with a NaOH solution and determining the pH of the equivalence point.

### Antioxidant capacity

The antioxidant activity was evaluated spectrophotometrically using the stable free radical DPPH test (1,1-diphenyl-2-picrylhydrazyl). The antioxidant activity was estimated using a standard ascorbic acid curve and the results are expressed as the equivalent percentage of ascorbic acid in terms of the DPPH consumed (% AAE: Vela et al. 2007).

### Vitamin C

Vitamin C was determined using the 2,6-dichloroindophenol titrimetric method (AOAC method for juices), which involves a redox titration with 2,6-dichloroindophenol (AOAC International 2005). The honey samples were prepared by dissolving 5 g of honey in 25 mL of 2 % oxalic acid and with folded filters filtration prior to the assessment (with 0.45 um cellulose acetate membrane filter). The vitamin C was quantified by RP-HPLC in isocratic mode, with a mobile phase of 0.01 % (v/v) H_2_SO_4_ (Panreac)/CTAB 0.01 M/MeOH 2 % (v/v) at pH 2.75 and 25 °C; a flow rate of 0.9 mL/min and with UV detection at 245 nm at 25 ± 1 °C (Vázquez-Odériz et al. 1994; León-Ruiz et al. 2011). The column used was a Lichrosorb RP-18 10 µm 150 mm × 4.0 mm (Merck), automatic injection system AS-2000, UV–Vis L-4250 model detector, interface D-6000. Standard solutions of vitamin C, were elaborated for the calibration curve prepared by dissolving 0.05 MHPO3.

Analytical were performed in triplicate for all parameters tested, except for pollen analysis. The pollen analysis was performed in duplicate and performed by two different experts, with an average variation less than 4 % in the global response on the main pollen type, which ensures a good correlation of the responses.

### Statistical analysis

In order to analyse the relationships of the distinct variables with the physicochemical and pollen data, the corresponding correlations and principal components analyses were carried out in order to see which influenced the segregation of the honey samples. These analyses were carried out with specific software, such as Biplot 1.1 (Smith and Lipkovich 1999–2002) and Olea-DP, working in Microsoft Excel (Martin Arroyo et al. 2013).

## Results

Each of the 70 honey samples were subjected to the specific analytical techniques to characterise the quality of the honey included in the D.O.P. regulations. The average proportion of pollen grains for the thyme honeys was 24 % (ranging from two honey samples with 18 % thymus pollen grains and one with 55 % thymus honey). The average pollen in honey from apiaries on limestone was 27.7 ± 11.91 % and on gypsum soils it was 28.5 ± 13.83 %. According to the Spanish Ministry of Agricultural and Food directive, thyme honeys must contain at least 15 % thyme grains, and a minimum of 15 % Thymus sp. pollen grains is necessary for this type of honey to be considered as monofloral thyme (Pérez-Arquillué et al. 1995; Caselles et al. 1998; Sáenz Laín and Gómez Ferreras 2000).

**Fig. 5:**
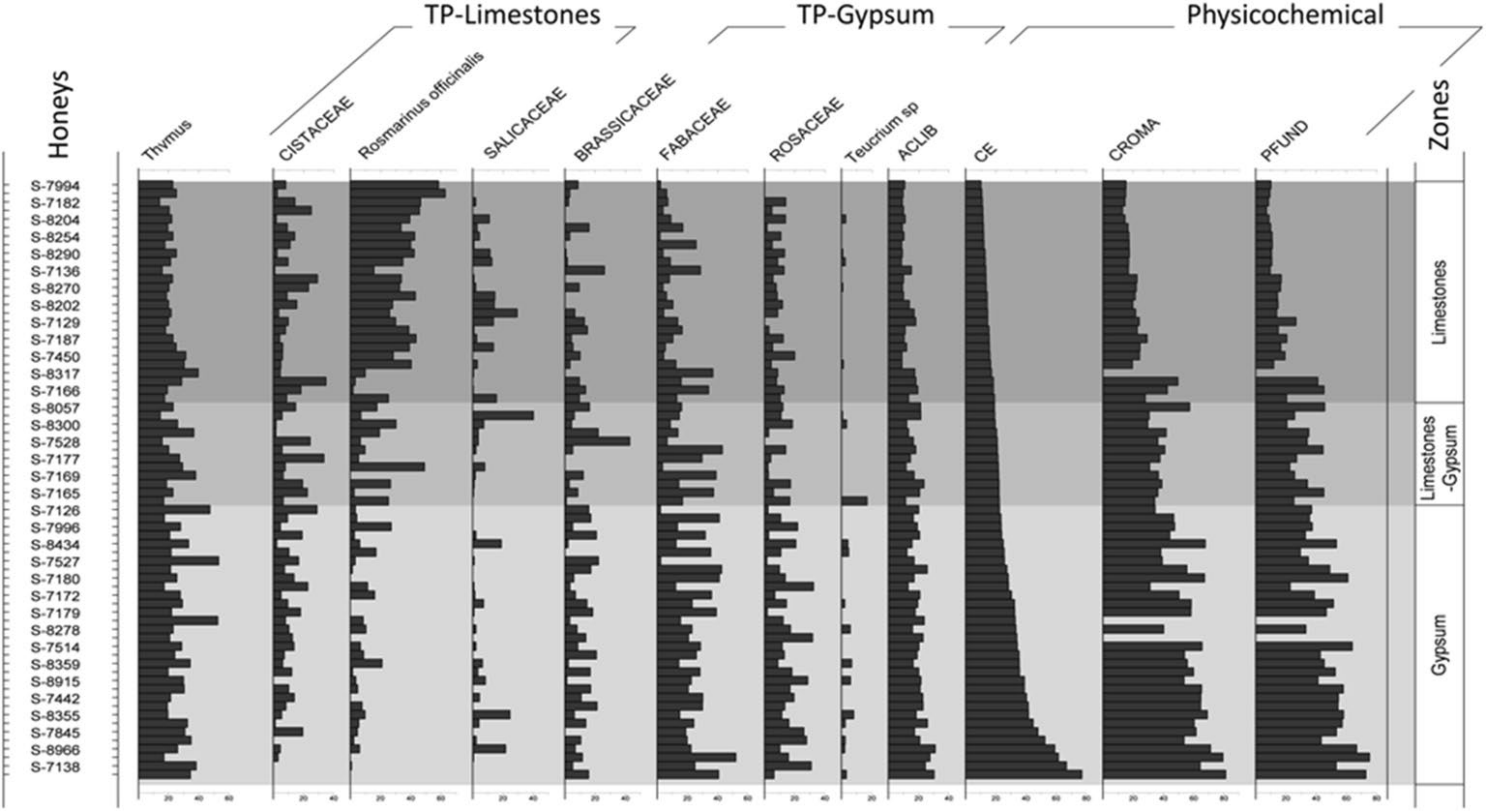
Pollen diversity in honeys samples

The pollen types in the honey samples were analysed and for each type of honey, we chose to represent the most significant taxa (at the family or genus level) for clarity. Anemophilous species of the Fagaceae family (*Quercus ilex* subsp. *ballota* (Desf.) Samp. and *Quercus faginea* Lam) were more strongly represented in the honeys on limestone. Indeed, these species form forests on limestone, whereas on the gypsum substrates in the area studied they are found as isolated individuals. In the honey from monofloral thyme isolated from apiaries located on gypsum, the families and genera best represented were Brassicaceae, Caryophyllaceae, Scrophulariaceae, Asteraceae, Fabaceae, Rosaceae and the genus *Helianthemum* (Cistaceae) and *Teucrium* (Lamiaceae). By contrast, in the honey from apiaries located on limestone the Salicaceae, Boraginaceae, Cistaceae, Fagaceae and *Rosmarinus officinalis* L (Lamiaceae) families predominate (Fig. 5; see full details in Additional file 1).

The degree of moisture was similar in all the samples, on average 16.50 % (with a standard deviation of 0.7). Indeed, the average moisture in honeys from limestone areas was 16.58 ± 1.631 % and in those from gypsum it was 16.52 ± 0.967 %.

**Fig. 6:**
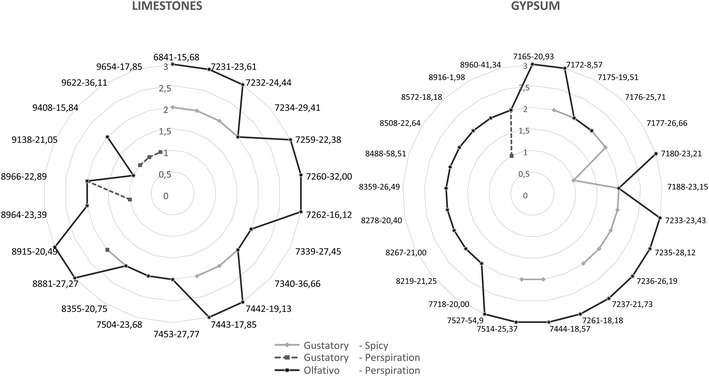
Representation of the organoleptic characteristics more representative of the two types of honeys from thyme studied

Among the physicochemical values analysed, the colour (Pfund), as well as the sensorial and organoleptic properties marked differences among the different monofloral thyme honeys. The panel of expert tasters classified the thyme honeys in two groups, in terms of colour, scent, aftertaste and texture. Gypsum thyme honey was dark, spicy and less dense, while that from limestone areas was clear. Basically, all honeys studied are classified within the Animal type and subtype Sweating, as olfactory quality. Moreover, this quality showed a degree of intensity of between 2 and 3 (high and very high). This corresponds organoleptically with the generality of honeys Spanish thyme. On the contrary, it is evident that within the taste characteristics, the honey of apiaries located in soils limestones have spicier flavors in honeys apiaries settled in soils gypsum, reaching high levels (2) this quality (Fig. 6).

The honey from the different substrates could also be segregated into two groups on the basis of conductivity, with a discrimination value of 0.3. We found that the thyme honeys with a conductivity was greater than 0.3 (0.57 average and 0.337 standard deviation) and that came from apiaries located on gypsum had a pH close to 4.7 ± 0.946, a free acidity of 24.15 ± 7.455 and a greater antioxidant capacity (91), as well as a higher vitamin C content and chroma values of about 60 (60.90 ± 20.893). Conversely, those with a conductivity below 0.3 (0.29 average and 0.064 standard deviation) and that came from apiaries at limestone sites had a pH around 4.3 ± 0.251, a free acidity of 18.21 ± 3.190, an antioxidant capacity of 60 and chroma of 40 (40.79 ± 21.190). Both groups of honey had similar amounts of glucose and fructose.

**Fig. 7:**
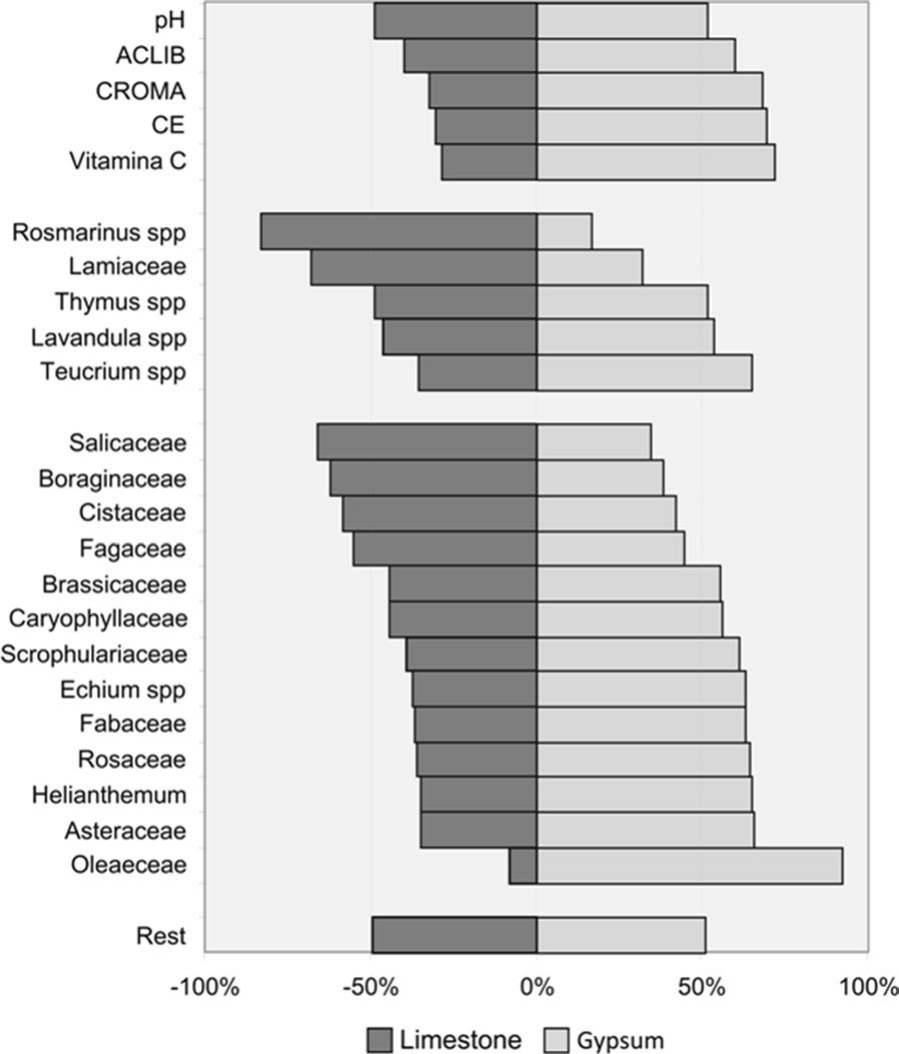
Representation of pollen types and physicochemical variables that determine both honey productions in the two productions of honey

Of the 27 physicochemical variables measured, those carried out on all the samples were selected for analysis taking into account the available relevant literature. Moreover, we considered the variables associated with a correlation coefficient greater than 70 % with conductivity. For variables that were virtually identical, such as the free acidity and antioxidant capacity, only one of them was analysed. In terms of the pollen spectrum, a selection was made on the basis of their presence in the different types of honey. Based on these considerations and after preliminary analysis, a total of 5 of physicochemical variables and 17 related to the nature of the pollen were used in the different analyses of the 70 samples. Using these variables produced a clear segregation of the honey samples into two groups (Fig. 7).

A segregation of the honey samples into two groups was also achieved by a multivariate analysis, the honey from the apiaries located on gypsum (32 samples) segregating to the right of the axis and those located in limestone to the left (25 samples). A few samples lay around the vertical axis (13) that were isolated from ambiguous territories. Indeed, apiaries located in areas of transition (Sacedón, Saceda, Trasierra, etc.) were included in this group.

After an analysis of correlation, we noted that chroma (CR), free acidity (ACLIB) and vitamin C are related to electrical conductivity (EC), with an index >70 %. To study the correlation between the pollen taxa and the EC, the variables with the greatest correlation index were selected. There were 8 pollen types that have a significant influence on the distinctive character of the honey, those of the genera *Thymus* sp, *Rosmarinus* sp. and *Teucrium* sp., and the families Cistaceae, Salicaceae, Brassicaceae, Fabaceae, Rosaceae.

**Fig. 8:**
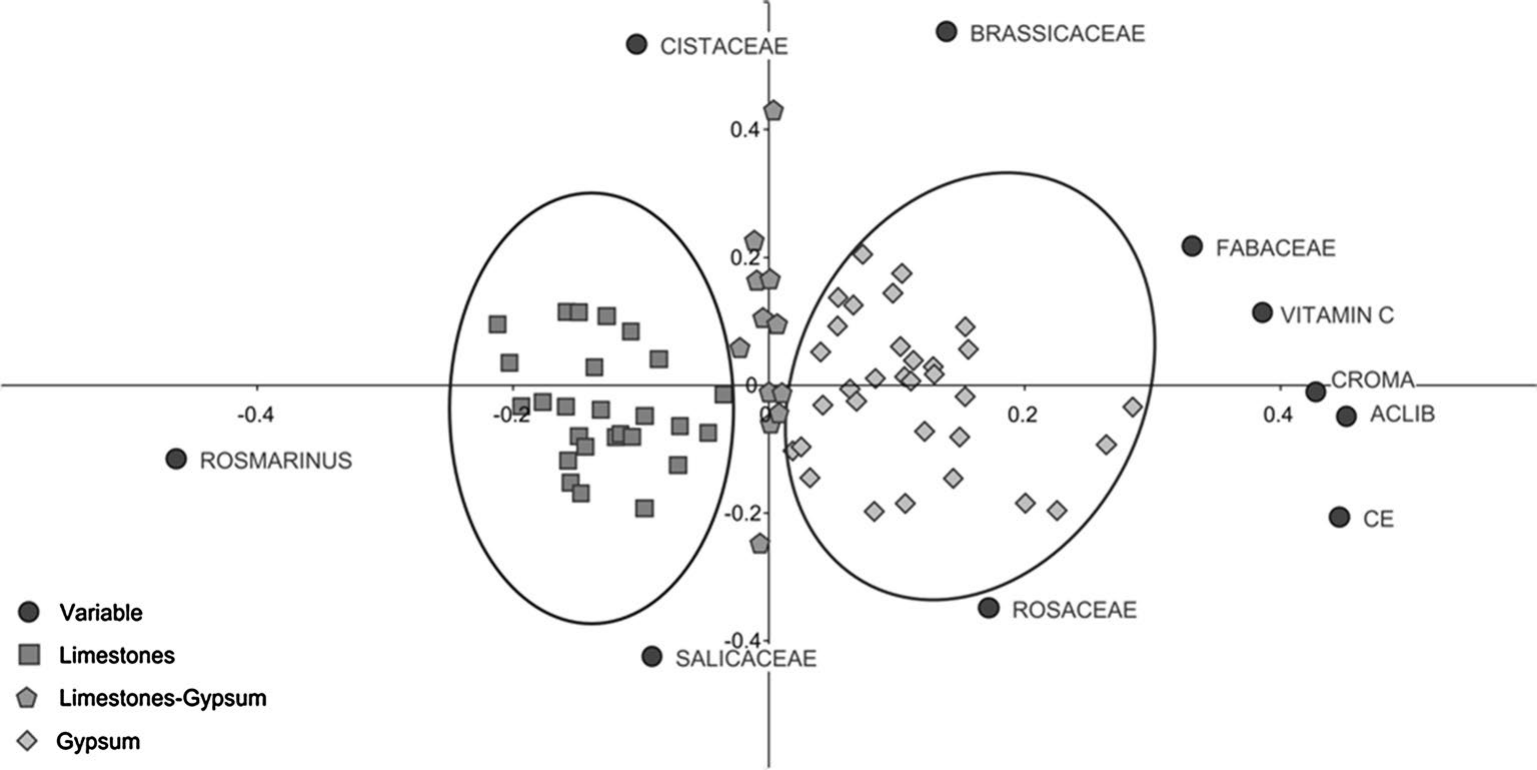
Distribution of the physico-chemical variables in the axis 1 and 2 of the principal components analysis. Segregation of samples according to the type of soil where apiaries are located

With the variables described above, a principal components analysis was carried out on the 70 samples studied using the Biplot 1.1 software package (Smith and Lipkovich 1999–2002), and following the centred and standardized variables method. Four axes were obtained with values >1 that express 73 % of the variance (Fig. 8).

## Discussion

Based on the differences detected in the sensory characteristics of the thyme monofloral honeys from the Baja Alcarria received at the Centro Agrario, we studied distinct physicochemical variables in these honeys. The moisture content of the honeys studied is similar because they come from the same geographical location and were gathered in spring (Fallico et al. 2004). Indeed, while the botanical resources available to produce the honeys may be different, the seasonal variability is similar. The apiaries located at higher altitudes (Vellisca, Saceda-Trasierra, Valdecolmenas and Abia de la Obispalia) have more moisture (17.25 %) than those located at a lower altitude (Pastrana 15.25 %, Horche 15.70 % or Utande 15.75 %). Only minor variability in the moisture of the honey would be expected if we take into account the changes in precipitation between May and June (5 l/m^2^ June) at the sites where the beehives are located.

When the parameters are analysed, segregation of the honeys into two groups was evident on the basis of conductivity with a discrimination value of 0.3. The electrical conductivity of the honey is closely related to the concentrations of mineral salts, organic acids and proteins, and it has proven to be useful to discriminate honeys of different floral origins (Mateo and Bosch-Reig 1998; Terrab et al. 2002a, b, 2004) and a good indicator of geographic origin (Anklam 1998; Acquarone 2004; Acquarone et al. 2007). Generally, a high conductivity is correlated with a high ash content and it corresponds to the darkest honeys. Here we observed different conductivity in the thyme monofloral honeys of the same geographic region but in areas with different substrates. Consequently, the physicochemical characteristics of the honeys are closely related to a specific floristic composition and soil type. This supports earlier conclusions from a global study of the minerals present in honey (Feller-Demalsy et al. 1989; Bogdanov et al. 2008), where good electrical conductivity was valued and the mineral content was related to the botanical origin, the soil-climatic conditions and the extraction techniques used to obtain the product. The honeys with the greatest conductivity correspond to apiaries located in gypsum soils, with specific and endemic vegetation (gypsophytes), while those with lower conductivity correspond to honey samples from apiaries located on limestone.

From a geological and biological point of view, it is evident that the chemical composition of gypsum (calcium sulphate and magnesium) increases conductivity. Gypsum soils contain water soluble salts and therefore, the Ca^2+^, Mg^2+^, Na^+^ and K^+^ present in the milieu when it rains are passed on to the water from the soil (favoured by a relatively warm environment) and these ions are incorporated into the plants through the roots. Gypsum soils also have an imbalance of nutrients, deficiencies in N and P, and an excess or imbalance in Ca and S, as well as a high Ca/Mg ratio (Gil Carrasco and Ramos Miras 2011). The gypsophytes are adapted to these environments and they can accumulate salts in vacuoles or excrete them through the leaves, glands or nectar (Cintrón et al. 1978; Ruiz et al. 2003; Merlo et al. 2011). The gypsum flora is specifically adapted to this medium and living in a salt-rich medium is the cause of the higher conductivity in the honeys that come from apiaries located on gypsum rather than on limestones (Grigore et al. 2011). In limestone soils, calcium carbonate is practically insoluble in water whereas its solubility is 100 times greater in gypsum soils (Gil Carrasco and Ramos Miras 2011). Thus, the calcium cations are immobilized in the clay fraction of limestone and therefore, it is more difficult for these cations to be passed on to the plants (Manresa 2005).

The pollen diagrams or the pollen loads of the honeys also segregates the samples into two groups, principally due to the floristic diversity of each substrate. All the honeys studied have an proportion of thyme pollen appropriate for them to be considered as monofloral (>15 % in D.O.P. La Alcarria honey, >13 % in Persano Oddo and Piro (2004) (Caselles et al. 1998; Persano Oddo and Piro 2004; DOCCM 05/08/2010; BOE No. 299 2010). There are multiple thyme species on gypsum soils, while *Thymus lacaitae* Pau is absent from limestone soils (Morales 2002; Morales 2010; Bartolomé et al. 2011a, b), which should therefore be better represented in the honey samples from gypsum. However, this did not appear to be the case since all the samples had a similar thyme pollen content. Thus, what seems to be most important to the bees is the abundance of the resource irrespective of the specific thyme species.

Based on the pollen data, we understand that the shrub structure is essential for the bees. Gypsum thickets are smaller, open shrubs where there is little overlap of the strata, while on limestone, the bushes have more strata and there is some overlap with others. The upper stratum could represent a screen that affects the pollination of plants that occupy the lower stratum. Hence, the weak overlapping of strata associated with gypsum shrubs might explain the greater diversity of the pollen in the honey from apiaries located on this type of substrate. The limestone bushes in this territory are rich in fruticose species of considerable height like: *Rosmarinus officinalis* and *Salvia lavandulifolia* Vahl. (Lamiaceae), *Lithodora fruticosa* (L.) Griseb. (Borraginaceae), *Cistus albidus* L (Cistaceae), *Staehelina dubia* L. (Compositae). Under their canopy, smaller species appear from several other families, such as *Thymus* sp., *Helianthemun* sp. and *Teucrium* sp. Within this group, rosemary is the largest species, with a long flowering period, followed by *Cistus*, *Lithodora* or *Salvia*. In the case of gypsum soils, the *Cistus* genus is absent and there is significantly less *Rosmarinus*, *Salvia* and *Lithodora* than on limestone soils, since these species are not gypsophytes and cannot live in an environment to which they are not adapted and that is toxic to them. On gypsum substrates and in the territories analysed, the following species appear that attain a similar size to rosemary: *Gypsophila bermejoi*, *Gypsophila pilosa*, *Gypsophila struthium* subsp. *struthium* (family Caryophyllaceae). These are strict gypsophytes with a relatively long flowering period, yet they form thickets in which there is virtually no strata overlap between the taller and smaller biotypes.

Since the shrubs on gypsum soils have a more open, steppe physiognomy, all species are visible and are likely to be visited/pollinated by bees, to which there is no physical impediment. Thus, in addition to the aforementioned representatives of the Caryophyllaceae family, other endemic species of this family, strict gypsophytes, can be pollinated, such as: *Arenaria cavanillesiana* and *Herniaria fruticosa* (all in the lowest stratum). This explains the abundance of this type of pollen in the honey. A similar situation occurs with other strict gypsophytes like *Helianthemun squamatum* and *Helianthemun conquense*, which are not unlike thyme and that form dense populations on soft gypsum substrates. By contrast, gypsum crusts or hard gypsum substrates represent an inhospitable medium for plant life but they are colonized by two species with special adaptations, *Teucrium pumilum* and *Herniaria fruticosa*. Finally, a few species within the Asteraceae family are also more abundant on saline and gypsum soils: *Centaurea hyssopifolia*, *Launaea fragilis, L. pumila, Senecio auricula* or *Artemisia herba*-*alba*. This gypsum-specific flora (Mota Poveda et al. 2011), with special adaptations to prosper in a restrictive environment, confers different properties to the honeys from thyme spp. compared to the honey from thyme that grows on limestone soil.

With respect to axis 2, a variation in the sample set conditioned by climate or soil moisture is evident. In honey from apiaries located close to soil water (slopes or valley bottom springs), there is a greater representation of pollen from the Salicaceae and Rosaceae families, whereas in those relying exclusively on rainwater for their water supply (climatic contribution), the Cistaceae or Brassicaceae families are better represented.

Regarding the sensory analysis, the lighter honeys have a lower ash content and weaker conductivity than the dark honeys (Terrab et al. 2003, 2004; Pérez et al. 2007; Vela et al. 2007; Bertoncelj et al. 2011; Gomes et al. 2010; Escuredo et al. 2011; Almeida-Muradian et al. 2013; Alqarni et al. 2014). In this study, lighter honeys obtained from apiaries located in limestone have on gustative level a feeling spicy higher. We obtained similar results, whereby mean Pfund values of 45 with a chroma of 60 correspond to honeys with high conductivity, dark honeys from apiaries located on gypsum, whereas the honey from apiaries located on limestone have weaker conductivity and Pfund values of 15, with a chroma of 21, corresponding to lighter honeys. The relationship between antioxidant capacity and polyphenols, pH, acidity, ash content or the conductivity of the honey (Pérez et al. 2007; Vela et al. 2007; Ciappini and Stoppani 2014; Jamroz et al. 2014) has been discussed elsewhere. A significant correlation between the total polyphenol content and the antioxidant capacity has been demonstrated, yet no correlation with the pH was observed (Pérez et al. 2007). Likewise, a linear relationship is established between polyphenols, conductivity and acidity. Here, we found a significant correlation between conductivity, free acidity, chroma and vitamin C.

As such, we conclude that honey from monofloral thyme located on gypsum soil has a greater antioxidant capacity, higher free acidity, a darker colour and greater pollen diversity than honey from thyme located on limestone. A linear correlation between antioxidant capacity and colour has been reported in the literature (Frankel et al. 1998; Ozcan and Olmez 2014). Not only has a dark colour been correlated with high antioxidant capacity (Gheldof and Engheseth 2002; McKibben and Engeshet 2002; Isla et al. 2011; Tezcan et al. 2011; Oliveira et al. 2012; Serem and Bester 2012; Sant’Ana et al. 2014; Canadanovic-Brunet et al. 2014; Kus et al. 2014) but the antioxidant capacity also apparently varies with the species composition and the season (Gheldof and Engheseth 2002; Isla et al. 2011; Rababah et al. 2014). We conclude that the darker honeys have a greater antioxidant capacity and correspond to monofloral thyme honey from apiaries located on gypsum soil. As the humidity and temperature are similar and the season of collection was spring, removal of these variables demonstrates the importance of soil composition on the honey properties. The antioxidant capacity of honey is similar to that of fruit and vegetables in terms of fresh weight, and it depends on the floral resources (Alvarez-Suarez et al. 2010), although soil type influences the variability and floristic diversity.

The two types of thyme honey studied show no significant difference in terms of sugar content or pH. An inverse relationship between sugar concentration and pH has been proposed (Pérez-Arquillué et al. 1995; Acquarone 2004; Rybak-Chmielewska et al. 2013), yet this could not be demonstrated here due to the limited variability of the sugars in the samples. The data presented in this study may be useful for beekeepers, promoting better marketing since a higher antioxidant capacity or higher vitamin C content may significantly affect the sales of a product given their added value from a nutritional point of view (Tomoi-Sato and Go-Miyata 2000). Hence, the location of the beehive may have an important influence on the marketing of the honey produced.

The Iberian gypsophilous vegetation is specifically preserved and prioritized by various global, European, national and regional guidelines, the most common of which is the Habitats Directive. Gypsum soils host a number of unique and singular endemic species that have led to the configuration of different figures aimed at the conservation of biodiversity in this area (Martín Herrero et al. 2003; Rivas Martínez and Penas 2003; Bartolomé et al. 2002, 2005, 2011a). Given the floristic singularity of the territory, bee pollination is very important to conserve biodiversity since it facilitates cross-fertilization and increases the genetic variability among populations Thus, the beehives established in such territories with a unique floristic richness provide added value from the point of view of conservation, as well as the interest aroused by the type of honey produced.

## Conclusions

Monofloral thyme honey from apiaries located in the same region, under similar climatic conditions but on different types of soil, has different pollen, as well as sensory and physicochemical properties. The soil type determines a specific floristic variability and this marks the properties of the honey. The honey from monofloral thyme from the Baja Alcarria region, characterized by pollen representative of the gypsophilous flora, has greater pollen variability and conductivity, and a higher antioxidant capacity, vitamin C content and acidity, and they are darker than the honeys from apiaries located on limestone with exclusively calcicolous flora. There is a correlation between conductivity, antioxidant capacity, colour and floristic composition.

The pollinizing action of bees is very important from the point of view of biodiversity conservation, providing an added value in terms of the conservation of the endemic gypsophilous flora of this region. Once the properties of these monofloral honeys has been detailed, the beekeepers may select the soil type on which to situate their apiaries depending on the characteristics of the honey sought.

## Additional file

10.1186/s40064-016-3243-9 Data on pollen spectrum of the thyme honey samples studied.

## Abbreviations

C.A.R. honey laboratory: Laboratorio de miel del Centro Apícola Regional
ICBB: International Commission for Bee Botany
PLSR: Partial Least Squares Regression
Pfund: Colour scale
CIELAB L*a*b* three-dimensional method: CIE color space and LABL* lightness, a* chromaticity +red/-green, b* chromaticity +yellow/-blue
C*ab: Chroma
hab: Tone
DPPH test: 1,1-diphenyl-2-picrylhydrazyl test
AAE: Ascorbic acid
AOAC: Association of Official Agricultural Chemists
RP-HPLC: Reversed phase High-performance liquid chromatography
D.O.P.: Denomination Origin Protected
CR: Chroma
ACLIB: Free acidity
EC: Electrical conductivity
DOCCM: Diario Oficial de la Comunidad de Castilla La-Mancha
BOE: Boletín Oficial del Estado
