# Supplementary material for: How soil type (gypsum or limestone) influences the properties and composition of thyme honey
Source: Springerplus. 2016 Sep 27;5(1):1663. doi: 10.1186/s40064-016-3243-9 (PMC5037098; doi:10.1186/s40064-016-3243-9)
Supplement: Supplementary file 1 — 10.1186/s40064-016-3243-9 Data on pollen spectrum of the thyme honey samples studied. [file 40064_2016_3243_MOESM1_ESM.pdf]

	72	C-5434	Y-6813	Y-6841	C-7102	C-7126	C-7129	C-7136	Y-7138	C-7165	C-7166	C-7167	C-7169	C-7170
	Muestras	5434	6813	6841	7102	7126	7129	7136	7138	7165	7166	7167	7169	7170
Suma	Tipo Polínico	C	Y	Y	C	C	C	C	Y	C	C	C	C	C
1,25	Alternaria													
10,0609802	Amariliaceae													
0,94340003	Anchusa													
1,14850998	Anthemis t_									0,41				0,74
3,12790996	Anthyllis t_													
46,5456195	Apiaceae	7,34	1,27	0,98	4,35							1,41		4,17
0,98039001	Artemisia													
2,82544997	Asphodelum													
88,8310702	Asteroideae	1,83		0,98	4,35									
101,54117	Boraginaceae		4,43			1,25	3,64		2,58				15,58	6,94
224,867338	Brassica sp_													
0,40485999	Buxus													
1,85185003	Calistegia													
2,4000001	Campanula spp_													
28,6930801	Campanulaceae		0,63						4,52					
19,988205	Carduus sp_							1,05		1,24	1,17			
1,24551001	Carex spp_													
18,8994898	Caryophyllaceae		1,90	0,98							0,60			
2,09338003	Castanea sativa Mill_											1,41		
31,5391203	Centaurea spp									1,55				
12,7444803	Chenopodiaceae													
1,49254	Chrozophora													
35,8040103	Cichorioideae (t_ Crepis)	1,83	0,63	1,96	4,35						1,80	0,70	1,30	
546,882676	Cistaceae	4,86	4,34	1,98	3,53	32,19	12,62	1,05		20,66	17,73	5,58	6,30	19,12
35,3373004	Cistus ladanifer L_		4,07	0,99										
2,12766004	Citrus spp_													
1,68427998	Colchicum													
1,07027996	Compositae													
1,68067002	Conium													
12,3673702	Convolvulaceae		0,63	0,98										
214,15606	Crataegus t_		7,59	2,94		2,50			5,16	2,33	7,78	7,75		2,78
519,874457	Cruciferae	1,83	11,39	10,78	6,52	17,50	16,36	26,32	4,52	7,75	13,17	4,93	11,69	20,83
3,13943005	Cucurbitaceae													
0,65807	Cyperaceae										0,33			
170,14249	Cytisus t_			0,50			0,97							
4,87804985	Diplotaxis spp_													
0,75187999	Dipsacaceae													
12,8316399	Dorycnium pentaphyllum Scop_													
219,873735	Echium spp_								2,58	21,71	12,57	3,52	1,30	2,78
8,5284901	Ericaceae												0,79	0,74
5,31690001	Esporas, hongos													
9,40506983	Eucalyptus spp_										1,20			
5,40652001	Euphorbiaceae			0,98										
3,84614992	Fabaceae													
54,5386704	Fraxinus			0,50										
0,94237	Fumaria										0,33			
43,8244796	Genista t_	2,78												
1,74685003	Geraniaceae													
3,27609	Hedera Helix													

Datos de Porto, A; Bartolome, C.; Martín T.

	72	C-5434	Y-6813	Y-6841	C-7102	C-7126	C-7129	C-7136	Y-7138	C-7165	C-7166	C-7167	C-7169	C-7170
	Muestras	5434	6813	6841	7102	7126	7129	7136	7138	7165	7166	7167	7169	7170
Suma	Tipo Polínico	C	Y	Y	C	C	C	C	Y	C	C	C	C	C
112,78491	Helianthemun													
40,4245992	Helianthus													
23,1659802	Heliotropium europaeum													
8,06451988	Hypecoum spp_													
10,1495097	Ilex aquifolim L_													
5,88234997	Inidentificable													
2,68198991	Inidentificado													
46,0698501	Juniperus			0,50		2,05		1,05	1,14	1,24	0,33	1,02	1,57	0,74
5,35637987	Labiatae													
42,4797802	Lamium													
93,1957	Lavandula latifolia Medik_	13,76	17,50											
18,8796599	Lavandula stoechas L_			1,96										
478,948792	Leguminosae	1,83	5,06	34,31	4,35	1,25	16,36	15,79	8,39	16,28	19,76	39,44	22,08	9,72
0,99010003	Ligustrum													
9,7662898	Liliaceae													
0,60241002	Linum													
2,04081988	Lithodora fruticosa													
8,54197985	Lonicera										0,60			
160,371649	Lotus t_			2,94		1,25		13,16	4,52		5,99		2,60	4,17
51,9872517	Lythrum spp_													
43,0417202	Medicago sp_	4,59							8,39		0,60		1,30	6,94
4,87804985	Melilotus spp													
33,4869501	Mentha													
2,32557988	Molinia													
172,39572	No aromáticas			1,96	8,70					0,78	0,60	0,70		1,39
187,122468	Olea europaea	1,39		0,99							1,34		7,87	13,24
25,2723101	Oleaceae													
37,6148894	Onobrychis sp									2,33	1,20			
3,36133999	Ononis t_			0,98										
23,8431096	Ornithopus T_												6,30	
77,5958197	Otros		2,44	0,50	2,35	0,68					0,33	1,02	7,09	0,74
36,7624197	Oxalis	2,78								7,02	11,71	0,51		
103,282345	Papaveraceae	7,64			9,41	2,74	3,88	20,00				1,52	0,79	0,74
2,92360008	Phlomis													
7,04337998	Pinus spp_	1,39												
3,24230009	Pistacia spp_													
26,0409101	Plantago spp_	4,59	0,63											1,39
7,01625004	Poaceae				2,35									
0,49505001	Polygalaceae													
24,6551996	Populus				22,35									
3,30882001	Prunus dulcis													
232,188105	Prunus t_	4,59		3,92	8,70			5,26					1,30	
785,654992	Quercus ilex		23,04	24,75	10,59	5,48	4,85	36,84	8,57	11,98	9,36	13,71	5,51	5,88
46,7800207	Quercus robur													
23,2048607	Quercus spp_													
262,601479	Quercus suber		0,54	1,52	8,24		4,85			0,41		1,02		
24,2629297	Ranunculaceae	1,83												
31,3128799	Raphanus													
10,5849398	Resedaceae													

Datos de Porto, A; Bartolome, C.; Martín T.

Suma	Tipo Polínico	C-5434	Y-6813	Y-6841	C-7102	C-7126	C-7129	C-7136	Y-7138	C-7165	C-7166	C-7167	C-7169	C-7170
	72 / Muestras	5434	6813	6841	7102	7126	7129	7136	7138	7165	7166	7167	7169	7170
		C	Y	Y	C	C	C	C	Y	C	C	C	C	C
2,73540998	Restos abeja	1,39												
2,19908997	Restos vegetales													
138,74148	Retama spp_		1,27							10,08		1,41	1,30	2,78
21,2478004	Rhamnaceae					0,68								
0,68493003	Robinia pseudoacacia													
149,289056	Rosaceae		3,80											
1034,42372	Rosmarinus officinalis L_	5,05			26,09	3,75	38,18	15,79	0,65	2,33	1,80	9,15		2,78
39,2931468	Rotos		1,08		5,88	0,68	0,97					0,51	1,57	2,21
11,3502301	Rubiaceae	[illegible]												
149,1375	Rubus	2,75	2,53	7,84				7,89	21,94	3,10	4,79	5,63		
11,25554	Rumex	1,83	[illegible]			6,25					0,60			
317,841069	Salix spp_		0,54	0,50	7,06		17,48			0,41	0,33	2,03	1,57	2,21
64,56865	Salvia spp_													
2,11837	Sanguisorba												1,30	
56,2793699	Satureja spp_		2,53											
3,46534991	Saxifraga													
5,33362007	Scandix													
133,0409	Scrophulariaceae	8,26			4,35	13,75			1,29	5,43	2,40	1,41		1,39
4,8040902	Senecio t_													
3,80777997	Sideritis	1,83												
0,76628	Silene t_													
2,16870004	Solanaceae													
29,1749697	Spartium													
71,0283697	Teucrium spp_													
1557,20445	Thymus	31,19	22,15	15,69	21,74	52,50	[illegible]	15,79	33,55	20,93	18,56	19,01	36,36	20,83
5,86044011	Tilia spp_													
154,038611	Trifolium t_		15,19	9,80					0,65	5,43	5,39		3,90	8,33
3,08658987	Ulmus													
5,34206009	Umbelliferae													
0,49505001	Urticaceae													
3,02423999	Veronica													
1,25	Viburnum													
53,6139696	Vicia t_													
14,4528501	Xanthium spp_								1,29		0,60			2,78
2,06383002	Zea mays													

Datos de Porto, A; Bartolome, C.; Martín T.

72	C-7172	C-7173	C-7174	C-7177	C-7179	C-7180	C-7215	Y-7442	Y-7443	C-7448	C-7450	C-7514	C-7527	C-7528
Muestras	7172	7173	7174	7177	7179	7180	7215	7442	7443	7448	7450	7514	7527	7528
Tipo Polínico	C	C	C	C	C	C	C	Y	Y	C	C	C	C	C
Alternaria														
Amariliaceae														
Anchusa														
Anthemis t_														
Anthyllis t_									0,98	1,05				
Apiaceae								2,61	7,14		1,10	1,49		
Artemisia									0,98					
Asphodelum														
Asteroideae				1,33			2,78				3,30		0,82	
Boraginaceae		6,67					8,33	5,22	1,79	2,08	9,89	7,46	0,82	1,37
Brassica sp_														
Buxus														
Calistegia														
Campanula spp_														
Campanulaceae														
Carduus sp_					0,76	0,60		0,56	0,98					
Carex spp_														
Caryophyllaceae									1,79	2,08	1,10			
Castanea sativa Mill_														
Centaurea spp				1,33								1,49		
Chenopodiaceae														
Chrozophora														
Cichorioideae (t_ Crepis)				2,67		1,79	2,78						0,82	
Cistaceae	5,41	8,47	5,26	32,80	16,67	12,50	2,38	12,43	7,84	8,95	4,76	12,15	17,31	29,75
Cistus ladanifer L_														
Citrus spp_														
Colchicum														
Compositae														
Conium														
Convolvulaceae														
Crataegus t_	4,76		22,22	1,33	1,69	3,57		1,74		12,50	15,38	10,45		
Cruciferae	7,14	33,33	16,67		16,95	5,36	2,78	9,57	19,64	4,17	7,69	7,46	22,95	52,05
Cucurbitaceae														
Cyperaceae														
Cytisus t_	1,35	3,39		1,06	0,76	1,79		2,61	1,79					1,27
Diplotaxis spp_														
Dipsacaceae														
Dorycnium pentaphyllum Scop_														
Echium spp_		3,33		22,67	23,73	17,86		2,61	1,79			7,46		
Ericaceae														
Esporas, hongos														
Eucalyptus spp_														1,37
Euphorbiaceae						3,57								
Fabaceae														
Fraxinus					0,76	0,60		0,56	1,96					1,27
Fumaria														
Genista t_														
Geraniaceae												0,93		
Hedera Helix														

Datos de Porto, A; Bartolome, C.; Martín T.

Datos de Porto, A; Bartolome, C.; Martin T.

72	C-7172	C-7173	C-7174	C-7177	C-7179	C-7180	C-7215	Y-7442	Y-7443	C-7448	C-7450	C-7514	C-7527	C-7528
Muestras	7172	7173	7174	7177	7179	7180	7215	7442	7443	7448	7450	7514	7527	7528
Tipo Polínico	C	C	C	C	C	C	C	Y	Y	C	C	C	C	C
Helianthemun														
Helianthus														
Heliotropium europaeum														
Hypecoum spp_														
Ilex aquifolim L_														
Inidentificable														
Inidentificado														
Juniperus	2,70													0,63
Labiatae														
Lamium														
Lavandula latifolia Medik_	2,38	3,33		6,67				0,87	1,79					4,11
Lavandula stoechas L_												1,49	2,46	
Leguminosae	30,95	16,67	5,56	22,67	15,25	30,36	5,56	11,30	16,07	2,08	2,20	14,93	2,46	4,11
Ligustrum														
Liliaceae														
Linum														
Lithodora fruticosa														
Lonicera														
Lotus t_					10,17	3,57		13,04	8,93	2,08		4,48		
Lythrum spp_														
Medicago sp_	2,38		11,11											
Melilotus spp														
Mentha														
Molinia							2,33							
No aromáticas	2,38									2,08		2,99		1,37
Olea europaea	14,86	23,73	22,81		15,91	32,14		2,82	1,96					
Oleaceae														
Onobrychis sp						1,79								
Ononis t_	2,38													
Ornithopus T_			17,54											
Otros	1,35	5,08	14,04	1,59	1,52	3,57		1,13	3,92	1,05	0,68	1,87	5,29	1,27
Oxalis		1,69		1,06	1,52	0,60						1,87		
Papaveraceae	2,70			3,70	0,76	1,19		0,56	0,98					0,63
Phlomis														
Pinus spp_														
Pistacia spp_														
Plantago spp_				1,33	1,69						1,10			
Poaceae														
Polygalaceae														
Populus														
Prunus dulcis														
Prunus t_								13,04	7,14					
Quercus ilex	12,16	3,39	7,02	15,87	11,36	8,93	1,19	7,91	20,59		1,02	10,82	10,58	5,06
Quercus robur														
Quercus spp_														
Quercus suber	1,35							0,56	1,96			1,87	2,88	3,80
Ranunculaceae														
Raphanus														
Resedaceae													2,88	

72	C-7172	C-7173	C-7174	C-7177	C-7179	C-7180	C-7215	Y-7442	Y-7443	C-7448	C-7450	C-7514	C-7527	C-7528
Muestras	7172	7173	7174	7177	7179	7180	7215	7442	7443	7448	7450	7514	7527	7528
Tipo Polínico	C	C	C	C	C	C	C	Y	Y	C	C	C	C	C
Restos abeja														
Restos vegetales														
Retama spp_				4,00										1,37
Rhamnaceae											0,34			
Robinia pseudoacacia														
Rosaceae				2,67		7,14			3,57					
Rosmarinus officinalis L_	16,67	3,33	5,56	5,33			55,56		7,14	31,25	21,98	5,97	3,28	8,22
Rotos		3,39	1,75	1,06		0,60	1,19	0,56	0,98	0,53	0,68			0,63
Rubiaceae														
Rubus	2,38	3,33				1,79		2,61	1,79				0,82	
Rumex												1,49		
Salix spp_	1,35				0,76			3,95		1,58		1,87	0,96	4,43
Salvia spp_														
Sanguisorba													0,82	
Satureja spp_								1,74						4,11
Saxifraga														
Scandix														
Scrophulariaceae								13,91	1,79	14,58	10,99	1,49	6,56	
Senecio t_														
Sideritis														
Silene t_														
Solanaceae														
Spartium														
Teucrium spp_														
Thymus	28,57	30,00	38,89	26,67	20,34	23,21	22,22	19,13	17,86	27,08	24,18	25,37	54,92	19,18
Tilia spp_														
Trifolium t_				1,33	10,17						1,10	5,97		1,37
Ulmus														
Umbelliferae														
Urticaceae														
Veronica														
Viburnum														
Vicia t_														
Xanthium spp_														1,37
Zea mays														

Datos de Porto, A.; Bartolome, C.; Martín T.

Datos de Porto, A.; Bartolome, C.; Martín T.

72	C-7718	Y-7845	C-7994	C-7996	C-7998	Y-8012	C-8057	C-8202	C-8204	C-8213	C-8231	C-8254	C-8258	C-8267
Muestras	7718	7845	7994	7996	7998	8012	8057	8202	8204	8213	8231	8254	8258	8267
Tipo Polínico	C	Y	C	C	C	Y	C	C	C	C	C	C	C	C
Alternaria														
Amariliaceae							0,78	0,63	0,97			1,08	4,45	
Anchusa														
Anthemis t_														
Anthyllis t_											1,09			
Apiaceae					2,59	0,63					2,40	0,56		1,00
Artemisia														
Asphodelum													0,40	
Asteroideae	1,33		1,14		0,86	4,38						1,69	3,42	
Boraginaceae			2,27			1,25			3,03	0,62			6,85	
Brassica sp_				4,88	20,69	1,88	12,77			6,79		2,82	1,37	4,00
Buxus													0,40	
Calistegia														
Campanula spp_											2,40			
Campanulaceae				1,63		0,63	4,26	0,75				0,56		
Carduus sp_	1,02		0,56	0,66	0,17		0,78	0,31			0,73			
Carex spp_												0,36		
Caryophyllaceae						2,50		1,50			0,80			
Castanea sativa Mill_													0,68	
Centaurea spp	4,00													
Chenopodiaceae	1,33				0,66									
Chrozophora														
Cichorioideae (t_ Crepis)					0,86			2,26			2,40			
Cistaceae	12,24	22,39	8,65	4,59	1,65	1,98	11,63	5,63	2,27	6,45	5,84	12,59	8,50	28,22
Cistus ladanifer L_								9,06			2,92			
Citrus spp_							2,13							
Colchicum							0,78							
Compositae														
Conium														
Convolvulaceae						1,25								
Crataegus t_		13,51						7,52	6,06		1,60			
Cruciferae	13,33		9,09											
Cucurbitaceae	1,33													
Cyperaceae									0,32					
Cytisus t_										1,23				
Diplotaxis spp_														
Dipsacaceae														
Dorycnium pentaphyllum Scop_														
Echium spp_				0,81				1,50					0,68	
Ericaceae													1,21	
Esporas, hongos														
Eucalyptus spp_														
Euphorbiaceae														
Fabaceae														
Fraxinus														
Fumaria														
Genista t_														
Geraniaceae					0,17				0,65					
Hedera Helix								1,88					0,40	

Datos de Porto, A.; Bartolome, C.; Martin T.

72	C-7718	Y-7845	C-7994	C-7996	C-7998	Y-8012	C-8057	C-8202	C-8204	C-8213	C-8231	C-8254	C-8258	C-8267
Muestras	7718	7845	7994	7996	7998	8012	8057	8202	8204	8213	8231	8254	8258	8267
Tipo Polínico	C	Y	C	C	C	Y	C	C	C	C	C	C	C	C
Helianthemun														12,38
Helianthus										2,47				
Heliotropium europaeum														
Hypecoum spp_														
Ilex aquifolim L_		2,70					7,45							
Inidentificable														
Inidentificado														
Juniperus				1,31				1,25	2,27	2,42	0,73	2,88	4,45	0,50
Labiatae				1,63		1,25								
Lamium						5,00		9,02		6,79	6,40			
Lavandula latifolia Medik_						3,13	12,77					1,13		
Lavandula stoechas L_	1,33													
Leguminosae	8,00	2,70		8,13	6,03	3,13		2,26						
Ligustrum														
Liliaceae														
Linum														
Lithodora fruticosa														
Lonicera	1,33					0,63								
Lotus t_	9,33	16,22	2,27			4,38	7,45							
Lythrum spp_														
Medicago sp_		2,70												
Melilotus spp				4,88										
Mentha					0,33		0,78		2,27		0,36	2,26		
Molinia														
No aromáticas				1,63	4,31	42,50		8,27	1,52		8,00	2,82		3,00
Olea europaea								0,31						
Oleaceae														
Onobrychis sp						1,25								
Ononis t_														
Ornithopus T_														
Otros	1,02	1,49				0,50		0,94				1,80		
Oxalis	3,06						1,55					0,36		
Papaveraceae	2,04	1,49		1,63		0,63		3,01			3,20			
Phlomis														
Pinus spp_					0,17		1,55	0,31	0,32		1,46			
Pistacia spp_						0,50								
Plantago spp_	1,33	5,41			1,72		2,13			1,23				
Poaceae										0,27			0,40	
Polygalaceae														0,50
Populus														
Prunus dulcis														
Prunus t_	29,33	8,11		3,25	0,86		6,38			5,88	3,20	7,34	4,11	38,00
Quercus ilex	2,04	14,93	0,38	17,38		5,45		5,94	11,00	3,23	15,69	[illegible]	3,24	1,98
Quercus robur														
Quercus spp_														
Quercus suber	2,04	4,48		26,56	8,91	8,42	5,43	18,75	21,68	7,26	9,49	[illegible]	[illegible]	3,47
Ranunculaceae												1,69	[illegible]	1,00
Raphanus														
Resedaceae														

72	C-7718	Y-7845	C-7994	C-7996	C-7998	Y-8012	C-8057	C-8202	C-8204	C-8213	C-8231	C-8254	C-8258	C-8267
Muestras	7718	7845	7994	7996	7998	8012	8057	8202	8204	8213	8231	8254	8258	8267
Tipo Polínico	C	Y	C	C	C	Y	C	C	C	C	C	C	C	C
Restos abeja												1,08		
Restos vegetales														
Retama spp_						8,75		6,77	8,33	9,88	4,80	1,69	3,42	6,00
Rhamnaceae					0,17				0,65	0,81		1,44		1,98
Robinia pseudoacacia													0,68	
Rosaceae		8,11		16,26			2,13	3,76	7,58	1,23	2,40	1,13	3,42	2,00
Rosmarinus officinalis L_		5,41	61,36	26,02	18,10		13,83	26,32	43,18	7,61	40,80	37,85	30,14	14,00
Rotos			0,38											
Rubiaceae										1,61	1,82	0,72	2,43	
Rubus	1,33			1,63	1,72	6,88	1,06		1,52	4,94	0,80	1,13		
Rumex														
Salix spp_					3,47	1,98		13,44	11,97	43,94	13,87	3,95	10,96	0,99
Salvia spp_							2,13	4,51	2,91		2,40	9,04	4,11	
Sanguisorba														
Satureja spp_										0,62		3,95	4,79	
Saxifraga														
Scandix														
Scrophulariaceae	4,00			0,66						3,09				
Senecio t_														
Sideritis							1,06							
Silene t_														
Solanaceae												0,72		
Spartium							2,13						3,42	1,00
Teucrium spp_									3,03	0,62			2,05	
Thymus	20,00	35,14	23,86	26,83	34,48	5,00	18,09	18,80	24,24	15,92	17,60	20,34	18,49	21,00
Tilia spp_														
Trifolium t_					6,90	2,50	3,19	0,75	1,52	4,94				8,00
Ulmus							2,33							
Umbelliferae								2,26		3,09				
Urticaceae														
Veronica														
Viburnum														
Vicia t_	2,67													
Xanthium spp_						2,50		0,75			0,80	0,36	1,21	
Zea mays							1,06							1,00

Datos de Porto, A.; Bartolome, C.; Martín T.

Datos de Porto, A.; Bartolome, C.; Martín T.

72	C-8270	C-8287	C-8290	C-8353	Y-8355	C-8356	C-8424	C-8428	C-8434	C-8506	C-8508	Y-8881	Y-8964	Y-8966
Muestras	8270	8287	8290	8353	8355	8356	8424	8428	8434	8506	8508	8881	8964	8966
Tipo Polínico	C	C	C	C	Y	C	C	C	C	C	C	Y	Y	Y
Alternaria														
Amariliaceae		0,90		0,86	0,40									
Anchusa					0,94									
Anthemis t_														
Anthyllis t_														
Apiaceae				1,35					1,94					
Artemisia														
Asphodelum												1,82		0,60
Asteroideae				1,35	4,72	0,76				0,85		0,91	0,58	
Boraginaceae						0,76			0,97					
Brassica sp_	10,53	9,09	0,77		6,60	0,76	5,94	2,65	0,97		2,83	8,18	9,94	6,02
Buxus														
Calistegia														
Campanula spp_														
Campanulaceae	2,63		3,57	1,35		0,76	2,97	2,65			0,94			
Carduus sp_					3,59							0,99		
Carex spp_						0,43								0,46
Caryophyllaceae													0,58	
Castanea sativa Mill_														
Centaurea spp						0,76					2,83			0,60
Chenopodiaceae									9,71					
Chrozophora														
Cichorioideae (t_ Crepis)									6,80	0,85			1,17	
Cistaceae	26,39	8,52	2,25	9,05	5,98	6,01	2,94	3,60	1,10	4,74				0,91
Cistus ladanifer L_								0,40		1,05			0,89	3,20
Citrus spp_														
Colchicum												0,91		
Compositae														
Conium														
Convolvulaceae							1,98		1,94		0,94	1,82		
Crataegus t_	2,63		10,71		4,72				4,85	0,85		7,27	5,85	1,20
Cruciferae														
Cucurbitaceae													0,89	0,91
Cyperaceae														
Cytisus t_	2,63			27,03		15,15		1,77			27,36	6,36	14,04	14,46
Diplotaxis spp_														
Dipsacaceae														
Dorycnium pentaphyllum Scop_												3,64		0,60
Echium spp_				4,05	0,94	3,79	0,99	8,85				2,73	16,96	3,01
Ericaceae									1,94				0,89	0,46
Esporas, hongos														
Eucalyptus spp_										6,84				
Euphorbiaceae										0,85				
Fabaceae														
Fraxinus				34,05								12,87	1,34	
Fumaria														
Genista t_														
Geraniaceae														
Hedera Helix														

Datos de Porto, A. Bartolome, C.; Martin T.

72	C-8270	C-8287	C-8290	C-8353	Y-8355	C-8356	C-8424	C-8428	C-8434	C-8506	C-8508	Y-8881	Y-8964	Y-8966
Muestras	8270	8287	8290	8353	8355	8356	8424	8428	8434	8506	8508	8881	8964	8966
Tipo Polínico	C	C	C	C	Y	C	C	C	C	C	C	Y	Y	Y
Helianthemun	12,50		5,86	1,29	3,59	1,29				2,11	3,16	4,95	1,34	0,46
Helianthus									32,69					
Heliotropium europaeum														
Hypecoum spp_														
Ilex aquifolim L_														
Inidentificable														
Inidentificado														
Juniperus							0,80	0,40	6,04					1,37
Labiatae														
Lamium														
Lavandula latifolia Medik_		0,83							1,94			1,82		2,41
Lavandula stoechas L_			0,71										0,58	1,20
Leguminosae								4,42	3,88					
Ligustrum														
Liliaceae						0,43							1,17	
Linum														0,60
Lithodora fruticosa														
Lonicera												1,82		
Lotus t_	1,75											1,82		0,60
Lythrum spp_			2,14											
Medicago sp_					0,94									
Melilotus spp														
Mentha	4,39	4,13				2,27		5,31	1,94		3,77	0,91	1,75	3,01
Molinia														
No aromáticas			2,86	2,70	15,09	3,03				3,42	1,89			1,20
Olea europaea												2,48	0,89	3,20
Oleaceae					0,80	1,29			1,65					
Onobrychis sp					0,94									
Ononis t_														
Ornithopus T_														
Otros						0,43								
Oxalis		0,45		0,43			0,27						0,45	
Papaveraceae			4,29		0,94	1,52			1,94			0,99	3,57	0,91
Phlomis					0,94		1,98							
Pinus spp_			0,45							0,53	0,35			
Pistacia spp_									2,75					
Plantago spp_	1,75							0,88						
Poaceae					0,80	0,43		0,80			0,35	0,50		
Polygalaceae														
Populus	1,85		0,45											
Prunus dulcis														
Prunus t_	6,14	5,79	0,71	2,70	5,66	8,33	2,97			0,85		10,00	5,85	6,63
Quercus ilex				8,19	2,79	15,45	54,28	33,60	12,64	11,58	46,67	9,90	7,59	7,76
Quercus robur										7,37	8,42	4,46		
Quercus spp_														
Quercus suber	4,17	9,42	15,77	2,59	3,98	11,16	12,03	9,60	9,34	4,74		2,97	1,79	4,11
Ranunculaceae	0,88	1,65	7,14				4,95	4,42						
Raphanus										1,71				
Resedaceae												6,36	0,58	

72	C-8270	C-8287	C-8290	C-8353	Y-8355	C-8356	C-8424	C-8428	C-8434	C-8506	C-8508	Y-8881	Y-8964	Y-8966
Muestras	8270	8287	8290	8353	8355	8356	8424	8428	8434	8506	8508	8881	8964	8966
Tipo Polínico	C	C	C	C	Y	C	C	C	C	C	C	Y	Y	Y
Restos abeja							0,27							
Restos vegetales			1,77			0,43								
Retama spp_		6,61			2,83						6,60			
Rhamnaceae	1,39			0,86		3,00						0,50	1,79	
Robinia pseudoacacia														
Rosaceae		4,13	0,71	6,76	1,89	6,82	2,97	3,54	2,91			3,64		
Rosmarinus officinalis L_	36,84	23,97	38,57	14,86	10,38	22,73	39,60	31,86	2,91	35,90	9,43	1,82	4,09	5,42
Rotos														
Rubiaceae						0,43	0,27				0,35			
Rubus									1,94			0,91		1,20
Rumex													0,45	
Salix spp_	0,46	14,88	9,91		26,29		1,34	2,40	8,79	5,79	2,46	1,82	2,92	19,28
Salvia spp_	1,75		0,71	4,05		2,27	6,93	4,42	0,97	10,26	2,83			
Sanguisorba														
Satureja spp_	4,39	6,61		4,05			8,91			12,82		0,91		
Saxifraga												3,47		
Scandix													2,92	2,41
Scrophulariaceae				2,70	2,33			0,88		0,85	13,21		2,34	
Senecio t_														0,60
Sideritis												0,91		
Silene t_														
Solanaceae														
Spartium		4,13	1,43	2,70	0,94					1,71				
Teucrium spp_	0,88		0,71	4,05	8,49	15,16		0,88	1,94		3,77	1,82	1,75	1,81
Thymus	22,81	16,53	22,86	18,92	20,75	15,15	19,80	23,89	15,53	21,37	22,64	27,27	23,39	22,89
Tilia spp_														
Trifolium t_		1,65	2,14	1,35	9,43			3,54	1,94	0,85		3,64	2,34	4,22
Ulmus								0,40						
Umbelliferae														
Urticaceae												0,50		
Veronica					0,40	1,72						0,91		
Viburnum														
Vicia t_													1,17	
Xanthium spp_	0,46								1,10	0,53	0,70			
Zea mays														

Datos de Porto, A; Bartolome, C.; Martín T.

72	Y-9138	Y-9332	C-9408	Y-9622	Y-9654	Y-9692	C-9746	C-9984	Y-9985	C-10013	Y-10112	C-10214	Y-10215	Y-10221
Muestras	9138	9332	9408	9622	9654	9692	9746	9984	9985	10013	10112	10214	10215	10221
Tipo Polínico	Y	Y	C	Y	Y	Y	C	C	Y	C	Y	C	Y	Y
Alternaria					1,25									
Amariliaceae														
Anchusa														
Anthemis t_														
Anthyllis t_														
Apiaceae					1,79	2,44								
Artemisia														
Asphodelum														
Asteroideae	4,51	1,63		2,78	5,36	17,07		2,44	8,33	0,38			1,52	2,75
Boraginaceae					3,57				4,17					
Brassica sp_	21,80	12,60	2,97	2,78	1,79	2,44	4,00	2,44	20,83		13,19			
Buxus														
Calistegia				1,85										
Campanula spp_														
Campanulaceae		0,84												
Carduus sp_		2,55	1,03											
Carex spp_														
Caryophyllaceae		0,84			1,79	2,44								
Castanea sativa Mill_														
Centaurea spp		8,40	0,99			7,32						1,10	0,61	0,55
Chenopodiaceae		0,84												
Chrozophora														
Cichorioideae (t_ Crepis)		0,84												
Cistaceae		1,27	1,55	7,41	1,25					3,70	8,43	0,78	2,72	9,03
Cistus ladanifer L_			4,12	1,85						1,73				5,05
Citrus spp_														
Colchicum														
Compositae											0,55	0,52		
Conium		1,68												
Convolvulaceae		0,84	1,98											
Crataegus t_		2,52	11,88	2,78	1,79		4,00	2,44	8,33	1,14				
Cruciferae										21,67		42,65	40,01	29,67
Cucurbitaceae														
Cyperaceae														
Cytisus t_	10,53	10,92	10,89		1,79			9,76		0,76				
Diplotaxis spp_						4,88								
Dipsacaceae	0,75													
Dorycnium pentaphyllum Scop_						2,44								
Echium spp_					10,71			4,88		6,84	0,55	0,37	4,23	
Ericaceae					2,50									
Esporas, hongos					2,50		2,82							
Eucalyptus spp_														
Euphorbiaceae														
Fabaceae											3,85			
Fraxinus		0,64												
Fumaria										0,25				0,36
Genista t_														
Geraniaceae														
Hedera Helix	0,48		0,52											

Datos de Porto, A; Bartolome, C.; Martín T.

72	Y-9138	Y-9332	C-9408	Y-9622	Y-9654	Y-9692	C-9746	C-9984	Y-9985	C-10013	Y-10112	C-10214	Y-10215	Y-10221
Muestras	9138	9332	9408	9622	9654	9692	9746	9984	9985	10013	10112	10214	10215	10221
Tipo Polínico	Y	Y	C	Y	Y	Y	C	C	Y	C	Y	C	Y	Y
Helianthemun		7,64	2,58	1,85	1,25	7,81	5,63	1,45		1,23		1,30	2,62	2,53
Helianthus	5,26													
Heliotropium europaeum		0,84				7,32					0,55		1,49	2,20
Hypecoum spp_														
Ilex aquifolim L_														
Inidentificable														
Inidentificado											2,68			
Juniperus	0,48			1,85	1,25			4,35		0,74				
Labiatae										1,14			1,34	
Lamium	3,76				1,79	2,44				0,76	3,30			
Lavandula latifolia Medik_		1,68	0,99			14,63						0,37		1,10
Lavandula stoechas L_			5,94		1,79								0,30	1,10
Leguminosae						2,44				11,03		9,19	11,08	31,87
Ligustrum			0,99											
Liliaceae	1,50				1,79			4,88						
Linum														
Lithodora fruticosa														
Lonicera									4,17					
Lotus t_	7,52	2,52		11,11	3,57	2,44			12,50					
Lythrum spp_		4,20					8,00						0,61	1,65
Medicago sp_						2,44					1,65			
Melilotus spp														
Mentha														
Molinia														
No aromáticas		7,56		5,56	5,36	4,88			4,17	0,76		2,21	1,34	15,38
Olea europaea	2,40	3,18	2,06				4,23	10,14						
Oleaceae				3,70	7,50					0,74	4,60	2,34	1,57	1,08
Onobrychis sp	0,75	0,84	25,74	2,78										
Ononis t_														
Ornithopus T_														
Otros				1,85	5,00	3,13		1,45						
Oxalis								1,45						
Papaveraceae			0,52			6,25		4,35		2,22		2,86	1,87	1,81
Phlomis														
Pinus spp_			0,52											
Pistacia spp_														
Plantago spp_		0,84												
Poaceae	0,48	0,64												
Polygalaceae														
Populus														
Prunus dulcis												3,31		
Prunus t_	4,51	4,20	1,98							7,98			5,91	
Quercus ilex	30,77	3,82	14,43	9,26	6,25	18,75	39,44	4,35	9,38	24,44			32,26	10,47
Quercus robur							5,63	5,80				15,10		
Quercus spp_											13,79			
Quercus suber	0,96		1,03											
Ranunculaceae														
Raphanus				5,56	8,93		4,00	7,32		3,80				
Resedaceae	0,75													

Datos de Porto, A.; Bartolome, C.; Martín T.

Datos de Porto, A; Bartolome, C.; Martín T.

72	Y-9138	Y-9332	C-9408	Y-9622	Y-9654	Y-9692	C-9746	C-9984	Y-9985	C-10013	Y-10112	C-10214	Y-10215	Y-10221
Muestras	9138	9332	9408	9622	9654	9692	9746	9984	9985	10013	10112	10214	10215	10221
Tipo Polínico	Y	Y	C	Y	Y	Y	C	C	Y	C	Y	C	Y	Y
Restos abeja														
Restos vegetales														
Retama spp_	0,75			5,56				4,88		2,28	29,12	3,68	3,05	
Rhamnaceae				1,85				5,80						
Robinia pseudoacacia														
Rosaceae								7,32	8,33	0,76	11,54	1,47	5,17	
Rosmarinus officinalis L_	2,26	1,68	1,98		10,71	2,44	40,00	4,88	12,50	23,57	2,20	28,68	7,07	8,24
Rotos												6,25	4,77	3,61
Rubiaceae	0,48	0,64	0,52											
Rubus	12,78	8,40	7,92		3,57	2,44		7,32		2,66		2,21	1,64	4,40
Rumex														
Salix spp_	0,75	4,20		5,56	1,79		4,00	29,27	4,17	3,80	1,65	2,21	1,22	
Salvia spp_		0,84	1,98			2,44								
Sanguisorba														
Satureja spp_		0,84												
Saxifraga														
Scandix														
Scrophulariaceae		1,68	1,98	2,78	3,57			4,88						
Senecio t_		4,20												
Sideritis														
Silene t_											0,77			
Solanaceae								1,45						
Spartium				2,78	8,93									
Teucrium spp_	0,75	1,68	0,99	5,56	1,79					1,52	4,40		5,84	
Thymus	21,05	5,88	15,84	36,11	17,86	17,07	36,00	2,44		3,42	27,47	2,57	13,98	1,10
Tilia spp_				5,56									0,30	
Trifolium t_		3,36	4,95	2,78				4,88		5,70				
Ulmus														0,36
Umbelliferae														
Urticaceae														
Veronica														
Viburnum					1,25									
Vicia t_		2,52							12,50					
Xanthium spp_														
Zea mays														

72	C-10387	Y-10627	Y-10656
Muestras	10387	10627	10656
Tipo Polínico	C	Y	Y
Alternaria			
Amariliaceae			
Anchusa			
Anthemis t_			
Anthyllis t_			
Apiaceae			
Artemisia			
Asphodelum			
Asteroideae		4,62	
Boraginaceae			
Brassica sp_	12,90	7,69	
Buxus			
Calistegia			
Campanula spp_			
Campanulaceae			
Carduus sp_		1,25	
Carex spp_			
Caryophyllaceae			
Castanea sativa Mill_			
Centaurea spp			
Chenopodiaceae			
Chrozophora			1,49
Cichorioideae (t_ Crepis)			
Cistaceae			
Cistus ladanifer L_			
Citrus spp_			
Colchicum			
Compositae			
Conium			
Convolvulaceae			
Crataegus t_			
Cruciferae			4,08
Cucurbitaceae			
Cyperaceae			
Cytisus t_			
Diplotaxis spp_			
Dipsacaceae			
Dorycnium pentaphyllum Scop_		6,15	
Echium spp_	1,61		22,45
Ericaceae			
Esporas, hongos			
Eucalyptus spp_			
Euphorbiaceae			
Fabaceae			
Fraxinus			
Fumaria			
Genista t_	14,52		26,53
Geraniaceae			
Hedera Helix			

Datos de Porto, A; Bartolome, C.; Martín T.

72	C-10387	Y-10627	Y-10656
Muestras	10387	10627	10656
Tipo Polínico	C	Y	Y
Helianthemun	11,76	8,75	7,46
Helianthus			
Heliotropium europaeum		10,77	
Hypecoum spp_	8,06		
Ilex aquifolim L_			
Inidentificable	5,88		
Inidentificado			
Juniperus			
Labiatae			
Lamium	3,23		
Lavandula latifolia Medik_			
Lavandula stoechas L_			
Leguminosae			
Ligustrum			
Liliaceae			
Linum			
Lithodora fruticosa			2,04
Lonicera			
Lotus t_			
Lythrum spp_		35,38	
Medicago sp_			
Melilotus spp			
Mentha			
Molinia			
No aromáticas			
Olea europaea		1,25	17,91
Oleaceae			
Onobrychis sp			
Ononis t_			
Ornithopus T_			
Otros		2,50	
Oxalis			
Papaveraceae			
Phlomis			
Pinus spp_			
Pistacia spp_			
Plantago spp_			
Poaceae			
Polygalaceae			
Populus			
Prunus dulcis			
Prunus t_		1,54	
Quercus ilex		5,00	
Quercus robur			
Quercus spp_	9,41		
Quercus suber			
Ranunculaceae			
Raphanus			
Resedaceae			

Datos de Porto, A; Bartolome, C.; Martín T.

72	C-10387	Y-10627	Y-10656
Muestras	10387	10627	10656
Tipo Polínico	C	Y	Y
Restos abeja			
Restos vegetales			
Retama spp_		1,54	
Rhamnaceae			
Robinia pseudoacacia			
Rosaceae	16,13		
Rosmarinus officinalis L_	11,29		4,08
Rotos			
Rubiaceae			
Rubus		1,54	
Rumex			
Salix spp_	11,29		
Salvia spp_			
Sanguisorba			
Satureja spp_			
Saxifraga			
Scandix			
Scrophulariaceae			
Senecio t_			
Sideritis			
Silene t_			
Solanaceae			
Spartium			
Teucrium spp_		1,54	
Thymus	16,13	16,92	18,37
Tilia spp_			
Trifolium t_	4,84		
Ulmus			
Umbelliferae			
Urticaceae			
Veronica			
Viburnum			
Vicia t_		12,31	22,45
Xanthium spp_			
Zea mays			

Datos de Porto, A; Bartolome, C.; Martín T.
